# Short-term effects of brief stair climbing interruptions on postprandial hyperglycemia during prolonged sitting: a randomized cross-over trial

**DOI:** 10.1038/s41598-024-77827-3

**Published:** 2025-01-17

**Authors:** Eswaran Thirunavukkarasu, Manaswi Reddy Aerva, Baskaran Chandrasekaran, G. Arun Maiya, Chythra R. Rao

**Affiliations:** 1 Department of Exercise and Sports Sciences, Manipal College of Health Professions, Manipal Academy of Higher Education, Manipal, Karnataka India; 2 Department of Physiotherapy, Manipal College of Health Professions, Manipal Academy of Higher Education, Manipal, Karnataka India; 3 Department of Community Medicine, Kasturba Medical College, Manipal, Manipal Academy of Higher Education, Manipal, Karnataka India

**Keywords:** Stair climb, Postprandial glucose, Attention, Prolonged sitting, Sustainability, Risk factors, Disease prevention, Occupational health

## Abstract

Prolonged sitting can negatively impact postprandial glucose levels and cognitive function. While short bouts of stair climbing are thought to mitigate these risks, the findings remain inconclusive. The present study aimed to explore the effects of stair climbing bouts on postprandial glucose and cognitive functions during prolonged sitting. Twenty-eight sedentary young adults (aged 20–30 years) underwent two intervention visits after standardised lunch for two hours: (1) STAIR: the participants climbed two flight of stairs for two minutes every 30 min; (2) SIT: the participants continued to sit. Blood glucose was measured using capillary finger prick method while attention function was measured using computer-based cognitive tests at baseline, end of 1st hour and 2nd hour. Significant interaction (F_2, 54_ = 15.96, *p* < 0.001) was observed for conditions and time. During STAIR visit, significant changes in postprandial glucose at 1st hour (β = − 2.6 mmol/dl, *p* < 0.001) and 2nd hour (β = 3.0 mmol/dl, *p* < 0.001). No significant difference in the attention functions with time and conditions was observed. Stair climbing interruptions may serve as a feasible and effective countermeasure to high glycaemic variability or excursions that occur during prolonged sitting after postprandial hyperglycaemia.

## Introduction

In the advent of newer information technologies and communication, a typical office worker spends significant proportion (60–80%) of his/her office work in sitting and majority of the sitting time is accrued in prolonged sitting bouts^1^. Prolonged sitting bout is now recognised as a pleiotropic risk factor for the majority of the cardiometabolic diseases through putative altered physiological mechanisms including high glycaemic excursion or variability, endothelial dysfunction and altered muscle metabolism^2,3^.

Ingestion of high glycaemic diets and prolonged sitting bouts postprandially are speculated to be the reasons behind periodic hikes in blood glucose levels which eventually increases risk of oxidative stress, inflammation, atherosclerosis and cardiovascular diseases^4^. Further this periodic rise in the postprandial glucose levels may alter blood brain barrier permeability^5^. Thus, compromised neural metabolism and neuroendocrine response may lead to cognitive dysfunction especially attention, mood and focus on the post lunch hours^6^. Hence it could be speculated that interrupting prolonged sitting bouts can offer protection against postprandial glycemia and cognitive dysfunction after lunch hours^7^.

For a decade, number of experimental trials have attempted to explore the effects of postprandial exercise on the glucose levels^8–13^. However, while these experimental trials have demonstrated the effects of the frequency and duration of exercise breaks, they provide limited information about the intensity of the exercise involved^10^. Moreover, the existing empirical evidence on the protective effects of physical activity breaks on glucose levels during postprandial sedentary periods remains mixed. While some studies have reported reductions in postprandial glucose levels and attenuated glycemic excursions following meals and periods of sedentary behavior^9,10,14^, others have found no significant changes in glucose levels after physical activity breaks during prolonged sedentary bouts^15^. This mixed effects may be probably due to the differences in the meal composition^15^ and the heterogeneity in the dimensions of physical activity breaks (frequency, intensity, type and duration)^14^ employed in the studies.

Low-intensity exercise breaks during prolonged sitting may not exert the same impact on muscle contraction mediated glucose transport, metabolism and insulin sensitivity as moderate-intensity breaks^16^. A fairly recent systematic review by Quan (2021) concludes that moderate-intensity exercise breaks are effective in reducing postprandial glycemia (standardized mean differences, SMD = -0.69, 95% confidence intervals, CI = − -1.00 to − 0.37) compared to light-intensity exercise breaks (SMD = − 0.46, 95% CI = − 0.66 to − 0.26)^14^. However, studies utilizing stair climbing as a moderate-intensity exercise break are still emerging.

Stair climbing (moderate-high intensity exercise) bouts are viewed as easy, simple and accessible in low-resource settings to foster euglycemic benefits by increasing both tonic and phasic muscle contraction mediated glucose transport and insulin activity^17^. Though early experimental trials have established the isolated effects of interrupting sitting with low intensity activity on the postprandial glucose levels^18^ and cognitive performance^19^ individually, the triangulated association between interrupting prolonged sitting with stair climbing bouts, changes in postprandial glucose levels and cognition is yet to be explored.

Thus, the research on moderate-high intensity exercise breaks such as stair climbing bouts on postprandial glucose is intriguing in sedentary workspaces where persistent attention functions are crucial for optimal workability and productivity^20^. Observational and experimental evidence also demonstrates a significant association between prolonged sitting time and the cognitive function including response inhibition, attention and information processing speed^21,22^. Hence, existing empirical evidence concludes that moderate-intensity exercise breaks are necessary to limit sedentary time in workplaces. Such breaks have the potential to improve plasma glucose levels and enhance attention functions, thereby fostering both health benefits and work productivity^19,23^.

Since the evidence claiming the health benefits of stair climbing especially on glucose levels after a meal and alteration in attention scores remain sparse, the present cross-over trial aimed to explore the effects of stair climbing interruptions on the blood glucose and attention levels after lunch hours in young adults.

## Results

**Fig. 1:**
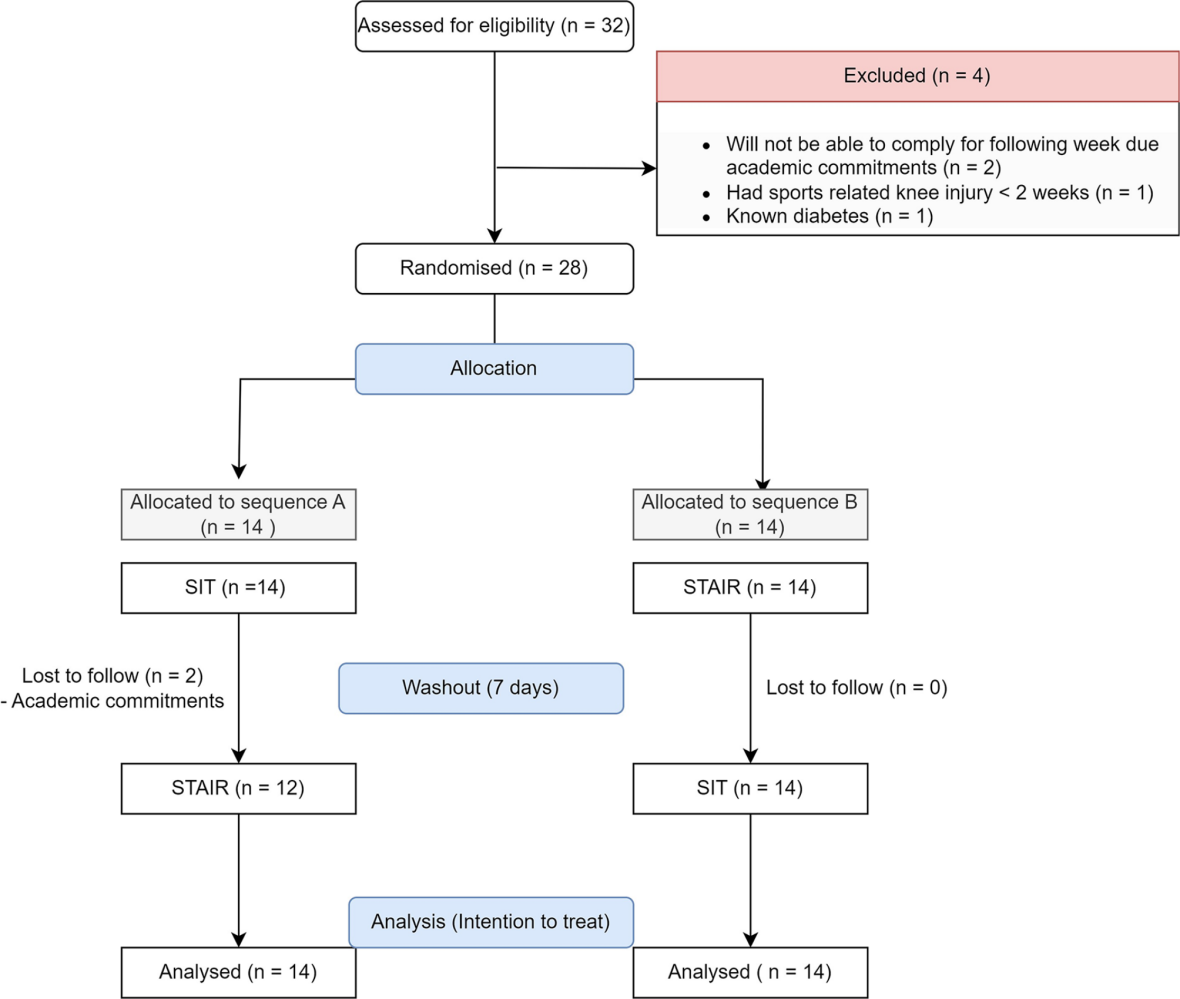
Participants screening, inclusion and analysis.

Twenty-eight participants were randomised in a counterbalanced manner to the sequence A (SIT as first visit, followed by STAIR at the next visit) or sequence B (STAIR as first visit followed by SIT at the next visit) interspersed by seven days. Twenty-six participants completed both the sequences while two participants missed the follow-up visit due to academic commitments. The flow of the participants screened, included and analysed is depicted in the Fig. 1.

### Baseline characteristics

Majority of the participants postgraduates with the median age of 24.1 ± 2.4 years and normal BMI (median = 22.8 kg/m^2^). The baseline postprandial glucose was 106.75 ± 18.75. Table 1 depicts the baseline characteristics of the participants included in the study.

**Table 1:** Baseline characteristics of the participants.

Baseline characteristics		Mean ± SD	
Age (years)		24.1 ± 2.4	
Body mass index (kg/m^2^)		22.8 ± 2.4	
Gender (women)		15 (53.7%)	
Physical activity level (MET hr per week)		583.5 ± 121.4	
Occupation	Postgraduate	20 (71.3%)	
	PhD scholar	6 (21.4%)	
	Academic staff	2 (7.1%)	

Variable	SIT	STAIR	p*
Blood glucose (mg/dl)	109.9 ± 10.7	104.7 ± 7.6	0.187
Simple reaction times (ms)	336.85 ± 53.82	330.83 ± 50.42	0.318
Choice reaction times (ms)	493.44 ± 88.28	478.39 ± 78.59	0.463

SIT trial visit when sitting was administered for two hours, STAIR trial day in which stair climbing bouts were administered for two minutes every 30 min of two hours of prolonged sitting.

*MET* metabolic equivalent, *SD* standard deviation.

*p value assessed from the one-way analysis of variance.

### Changes in the outcomes with interventions

#### Glucose levels

**Fig. 2:**
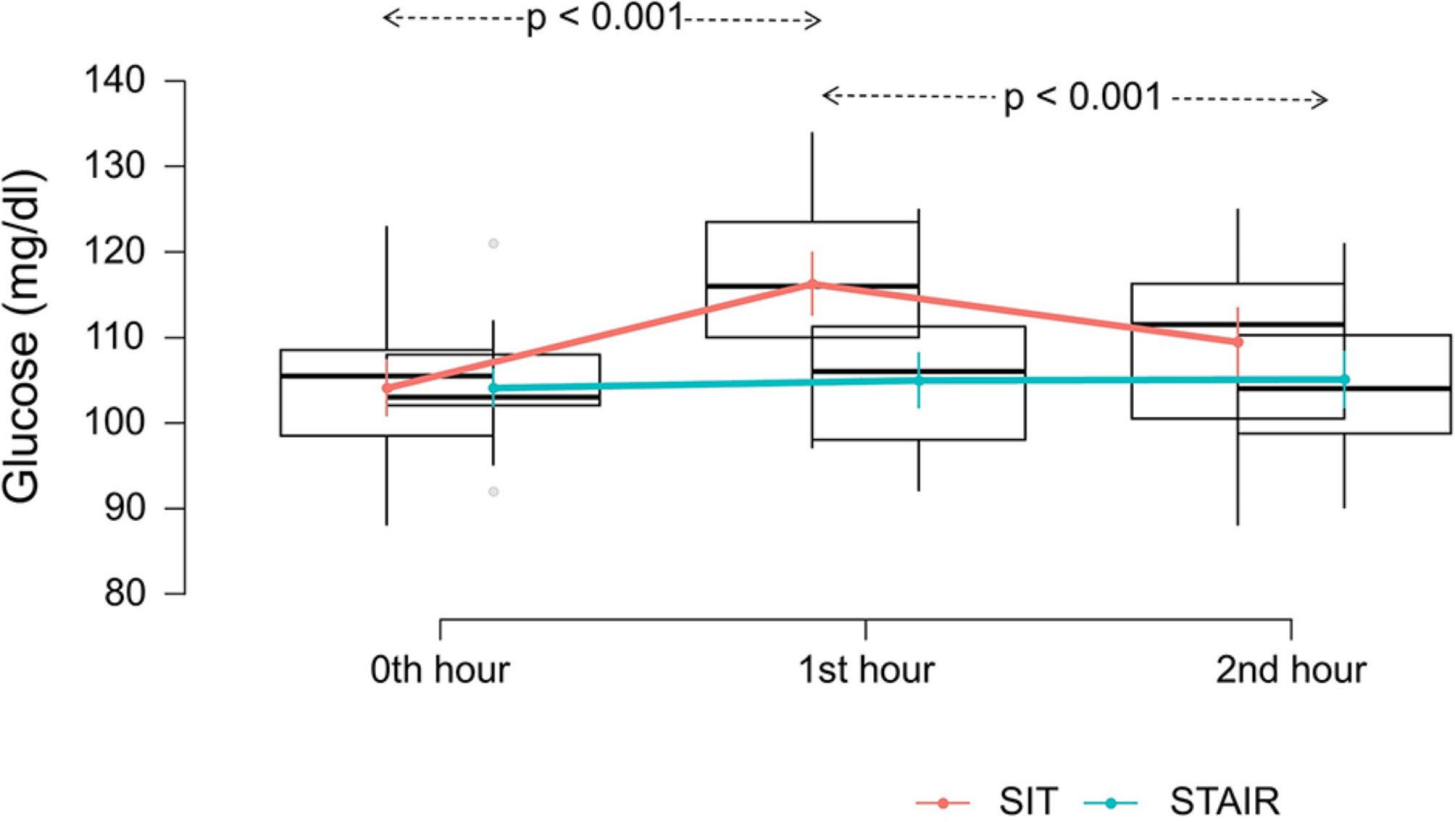
Blood glucose levels across time (0th, 1st, 3rd hour) and condition (SIT and STAIR). *p* < 0.05 indicates levels of significance. Box plots represent the estimated marginal means of glucose levels with standard error bars. Red line represent median change in the blood glucose levels during the SIT visit while green line represent median change in the blood glucose levels during the STAIR visit.

Significant interaction (F_2, 54_ = 15.96, *p* < 0.001) was observed for conditions (SIT, STAIR) and time (0th, 1st and 2nd hour). Similarly, main effects for condition (F_1,27_ = 11.81, *p* = 0.002) and time (F_2, 27_ = 13.27, *p* < 0.001) were statistically significant. Fixed effects estimation revealed that STAIR visit had significant changes in postprandial glucose at 1st hour (β = − 2.6, *p* < 0.001) and 2nd hour (β = 3.0, *p* < 0.001) compared to SIT visit and baseline. Figure 2 depicts the postprandial glycaemic changes across time and conditions.

#### Attention functions

**Fig. 3:**
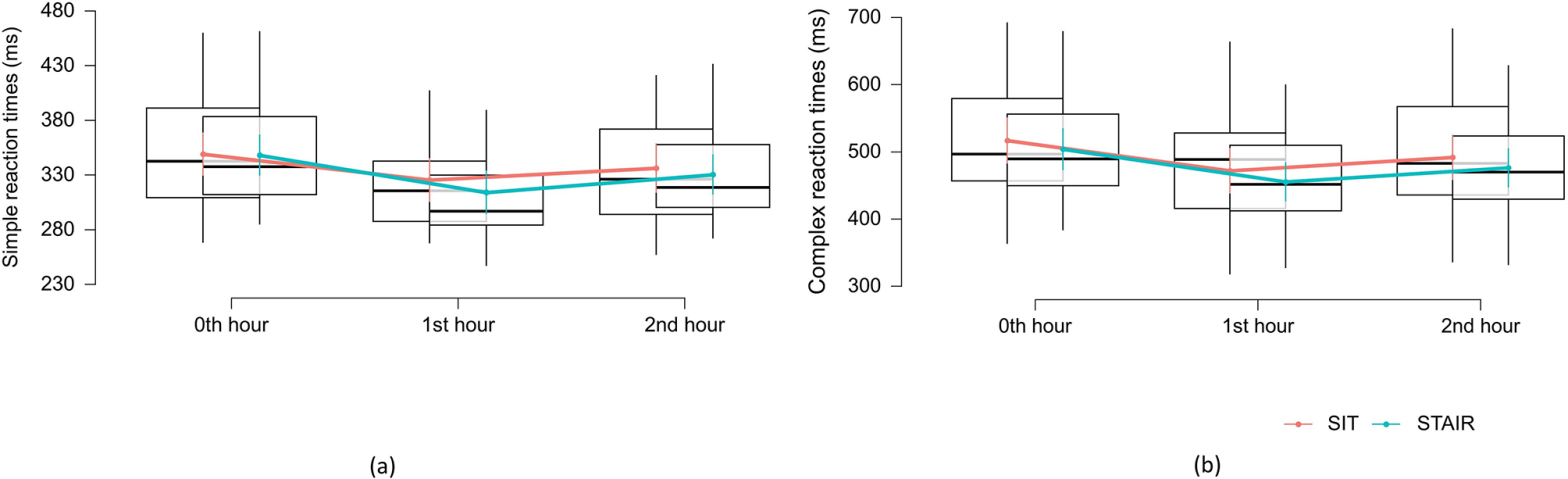
Reaction times to Deary-Liewald’s simple reaction tests (**a**) and Choice reaction tests (**b**) across time and condition. *p* < 0.05 indicates levels of significance. Box plots represent the estimated marginal means of reaction times (ms) with standard error bars. Red line represent median change in the reaction times during the SIT visit while green line represent median change in the reaction times during the STAIR visit.

We did not find any significant difference in either simple reaction or choice reaction times with time and conditions. While no interaction effects were evident, the main effects for time were statistically significant for both simple reaction times (F_2, 94.15_ = 33.18, *p* < 0.001) and choice reaction times (F_2, 39.26_ = 87.01, *p* < 0.001). Further main effects were significant for STAIR interventions in choice reaction times (F_1, 27.05_ = 12.75, *p* = 0.001). Fixed effect estimation revealed a positive effect at 1st hour for simple reaction (β = 14.71 ms, *p* < 0.001) and choice reaction (β = 24.43, *p* < 0.001), while negative effect at 2nd hour for simple reaction (β = − 14.15, *p* < 0.001) and choice reaction (β = −22.47, *p* < 0.001). STAIR visit revealed a positive effect of choice reaction times (β = 7.53, *p* < 0.001) than SIT interventions (Fig. 3).

### Association between mean change in postprandial glucose and cognition

**Fig. 4:**
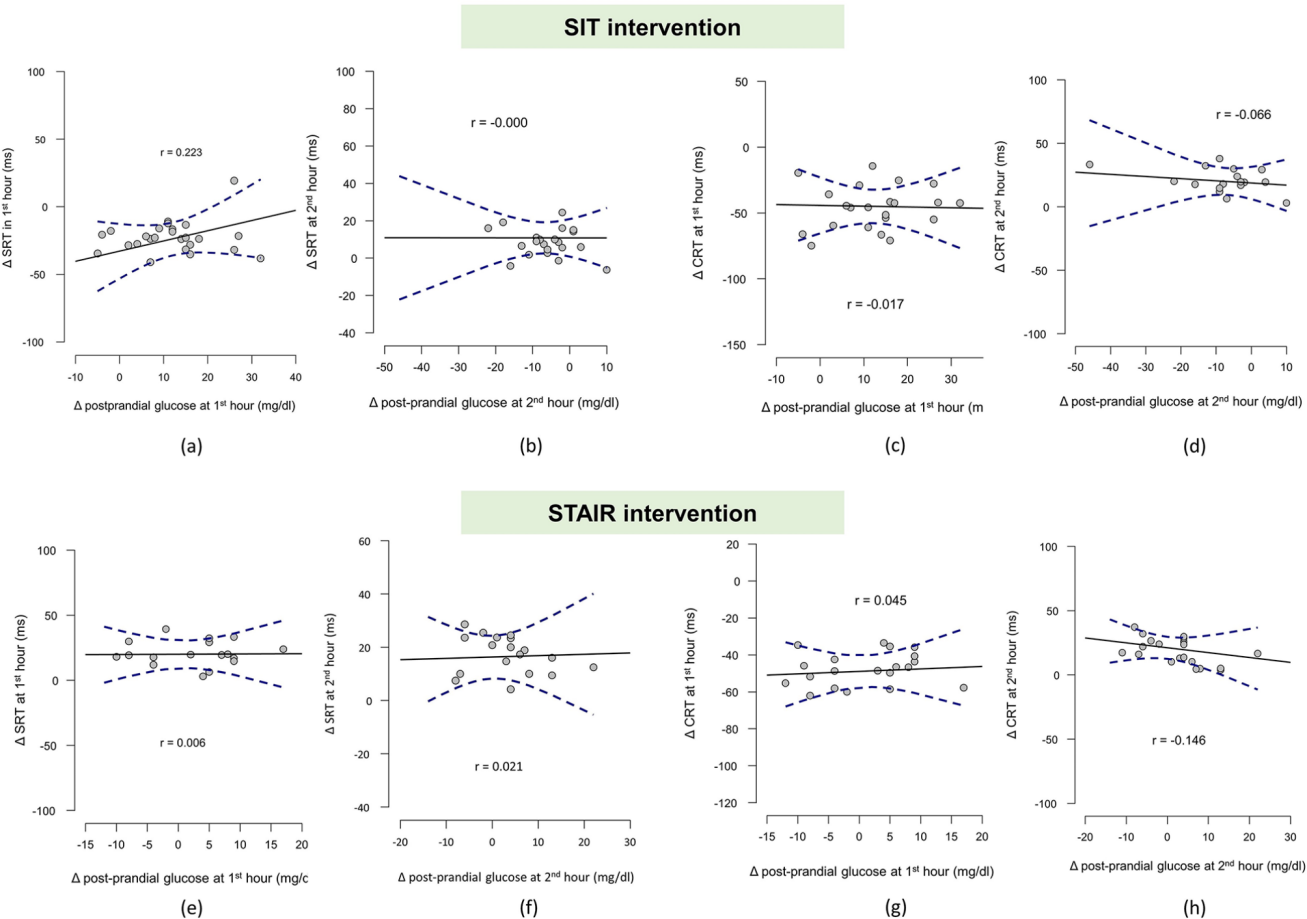
Scatter plots with linear regression lines and confidence intervals showing the relation of mean change in the postprandial glucose levels with the mean change in the cognitive functions. While (**a**) and (**b**) depicts the relation between change in glucose and attention to simple reaction stimuli at 1st and 2nd hour respectively, (**c**) and (**d**) illustrates the relation between change in glucose and attention to choice reaction stimuli at 1st and 2nd hour respectively during SIT visit. Similarly (**e**), (**f**), (**g**) and (**h**) illustrates relation between glucose and attention to simple and choice reaction stimuli during STAIR visit.

No significant relation between the mean change in the glucose levels and mean change in simple reaction times and choice reaction times with time and conditions. Figure 4 depicts the relation between the change in glucose and attention functions with time and conditions.

## Discussion

Our research aimed to explore the potential of stair climbing as a post-meal activity on blood glucose levels and attention functions. Our research findings revealed the significant benefits of stair climbing interventions in mitigating postprandial hyperglycaemia associated with the post lunch sedentary time. However, our study did not find favourable effects of the stair climbing on cognitive functions.

Postprandial hyperglycaemia^24^ and prolonged sitting bouts^25^ are found to be two distinct risk factors for the cardiovascular diseases. Previous systematic reviews consistently showed that periodic interruptions to the prolonged sitting postprandially are speculated to offer potential cardiometabolic health benefits by reducing postprandial glycemia and its excursions^26,27^. Studies have administered standing, walking and dynamic workstations with limited studies exploring the viable options of moderate exercise such as stair climbing interventions^28,29^. Systematic reviews have consistently confirmed that postprandial glucose can be significantly reduced with moderate-intensity exercises compared to light-intensity activities such as standing and walking^30^. Our findings further strengthen the existing literature that moderate intensity exercise may be better than light intensity exercise in mitigating the postprandial glucose levels^14^. Further, our study can pave the way for longitudinal studies to explore the cardiometabolic benefits of stair climbing as a habit.

In our study, the increased postprandial glucose levels following postprandial sedentary behavior are found to be attenuated with stair climbing bouts during the 1st and 2nd hour consistently. Our study findings are consistent with the systematic reviews which confirm that moderate intensity exercise breaks such as stair climbing interventions post lunch hours could attenuate the postprandial glycaemic variability or excursion, eventually preventing from cardiometabolic disease risk^31^. Our study findings align with recent experimental studies that concluded moderate-intensity physical activity breaks can reduce postprandial hyperglycaemia^18,20^.

Our study findings indicate that the postprandial glucose levels peak by 2.6 mg/dl at the end of 1st hour and drops back to the pre-prandial levels by the end of 2nd hour. This contrasts with the findings of Solomon (2020), who demonstrated that glucose peaks 35–40 min after breakfast^32^. The potential reasons behind the late onset of rise in postprandial glucose levels may be due to following reasons: (1) we administered a typical solid Indian lunch, unlike the liquid breakfast offered by Solomon (2020), which might have caused a slower increase in blood glucose levels^32^; (2) studies have measured the blood glucose levels continuously while our study used point care devices at only three time points (0th, 1st and 2nd hour); (3) difference in the intensity and timing of the exercises administered postprandially. The same study also recommends exercise breaks should be administered at 30 min post lunch so fuel can be used directly from the food ingested rather than hepatic glucose^32^. Our findings also confirms that the rise in postprandial glucose can be mitigated when the exercise administered at 30–40 min post lunch.

Cognitive dysfunction (longer reaction times to the cognitive load) post lunch is commonly debated. While few studies found postprandial high glycaemic index favour attention and recall^33^, few found excessive postprandial glycaemic excursions to be associated with the derangement of executive and attention functions^34^. Our findings demonstrate that physical activity breaks may not have favourable cognitive outcomes which concur with the previous studies^15,35,36^. It can be argued that the cognitive functions especially attention can be mediated by sleep, composition of the diet and the intrinsic motivation to the workload (office tasks). Further, no significant association between the change in the postprandial glucose levels and the attention scores at different time points was found in our study. Long term randomised controlled studies controlling other confounding factors of sleep, mood and similar office tasks are warranted.

The major strengths of the study are: (1) the robust design of the randomized crossover trial with the standardization of simulated office tasks, and (2) the exploration of stair-climbing interventions, which are easy, readily available, and accessible for individuals in low- to middle-income countries. Few limitations worth mentioning are: (1) the administration of a shorter time frame of prolonged sitting (120 min) and its interruptions contrasts with the majority of studies, which have administered prolonged sitting durations ranging from 150 to 540 min^14^. This may potentially limit the ecological validity of the study; (2) administered in a controlled laboratory setting, which manages potential confounders such as movement and standardization of computer tasks, may lack generalizability to naturalistic office settings that may involve other outcome-modifying factors, including psychology, diet, workload, and peer relations; (3) In the current study, only the acute effects of stair climbing were explored, while the long-term effects of integrating stair climbing bouts into real-world scenarios may differ and should be further investigated^29^; (4) the glucose levels blood glucose levels were measured using capillary blood glucose which are known to affected by heat and humidity and questioned for its validity^37^; (5) The present study assessed single domain of the multivariate cognitive functions such as focus, problem solving, executive function, language and visuospatial skills. Further studies advocating multivariate cognitive battery are warranted.

## Conclusion

Stair climbing interruptions may serve as a feasible and effective countermeasure to high glycaemic variability or excursions that occur during prolonged sitting after postprandial hyperglycaemia. Future long-term studies are warranted to further substantiate the benefits of stair climbing in mitigating postprandial hyperglycaemia in naturalistic settings during sedentary behavior.

## Methods

The present randomised counterbalanced trial is reported as per CONSORT 2010: extension to randomised cross-over trials. The checklist is attached as supplementary table S1. The study was approved by Institutional Ethics Committee (IEC 263/2023) on 18th November 2023 and prospectively registered in the Clinical Trial Registry of India (CTRI/2024/02/062247) on 5th February 2024. The study was conducted between February – May 2024. The research was administered as per the general set of principles laid by Declaration of Helsinki on ethical principles of human Research^38^.

### Study design

The present randomized crossover study aimed at investigating the impact of stairclimbing interruptions on postprandial glucose levels and attention functions during two hours of extended sitting among sedentary young adults. Participants completed two 2-hr interventions randomized as follows: (1) prolonged sitting with and (2) without stair climbing interruptions every two minutes for 30 min in a counterbalanced order with a washout period of at least six days between visits. The sequence in which the participants received their intervention was determined by random assignment.

### Participants

Young adults with the age of 20–30 years, both genders, with the self-reported daily sitting time of six hours per day and with either educational or occupational screening time of six hours per day were eligible for the study. Further, the participants with the history of diabetes, altered fasting glucose levels, current smoker (< 6 months), altered chronic neurological and musculoskeletal disorders that would limit optimum participation in stair climbing interventions or cognitive disorders with a potential limitation to perform cognitive tests and pregnant were excluded from the study. Nevertheless, the participants with the prick allergy, major illness/injury (acute or chronic), without any cardiovascular disease, or psychiatric or neurological disorder or taking antipsychotic drugs which may affect their cognition were also excluded from the trial.

### Sample size

To find an assumed reduction (due to young healthy adults) in the postprandial glucose (-1.6 mg/dl) at the end of 1st hour (the previous experimental trial has found − 3.6 mg/dl at the end of 45th minute of 3 min-stair climbing bouts^39^) with a moderate effect size (d = 0.57), 27 participants were needed at a power of 80% (1-β = 0.80) and 5% significance (α = 0.05). Sample size was estimated under F test family and statistical test: ANOVA-fixed effects, special, main effects and interactions (as we are unsure about the homogenous baseline values after six days washout) with the effect size of 0.57, numerator df = 1 and number of groups = 2. The sample size was estimated using G*Power software (version 3.1.9.6, University of Kiel, Germany).

### Procedure

Young adults who had a self-reported daily sitting time for more than six hours per day at a multifaceted university, were invited to participate through flyers displayed on university and hostel noticeboards, as well as on the social media pages of the university student trading chambers. After written informed consent, the volunteers were recruited based on the eligibility criteria mentioned above. The eligible participants visited the central testing facility thrice: (1) *familiarisation day*: In this visit, the participants demographic and anthropometric data were collected. The participants were explained about study procedure and familiarised with the two hours computer-based text copying, typing, logical reasoning and drawing tasks for ten minutes. Also, they were familiarised with stair climbing for one flight and computer-based choice reaction tests. The participants were randomised order of the interventions (counterbalanced) using chit method and asked to pick up one among 28 chits having two equal intervention sequences: (a) SIT-STAIR – prolonged sitting during the first visit followed by the stair climbing on the next intervention visit; (b) STAIR-SIT – stair climbing intervention visit followed by the prolonged sitting visit interspersed by a period of minimum of six-day wash-out period. Once assigned, the chit was discarded, and the other participants were asked to choose from the remaining chits. After a washout period of six days, the participants underwent the next intervention. The participants were instructed to carryout regular routine work and not to start any strenuous physical activity (e.g. gym) for next 8–10 days. The participants were instructed to have at least 7–8 h of undisturbed sleep and standard dinner 33% total calories of day (carbohydrates – 51%, protein – 18%, and fat − 31%) the previous day of two intervention visits. Further participants were instructed to have standardised breakfast (33% of the total daily calories) and no strenuous physical activity on the day of testing was encouraged during the days of intervention visits. On the intervention day, the participants were requested to visit the lab at 1:00 PM in a fasted state (without having lunch and a gap of 5 h from the time of breakfast).

**Fig. 5:**
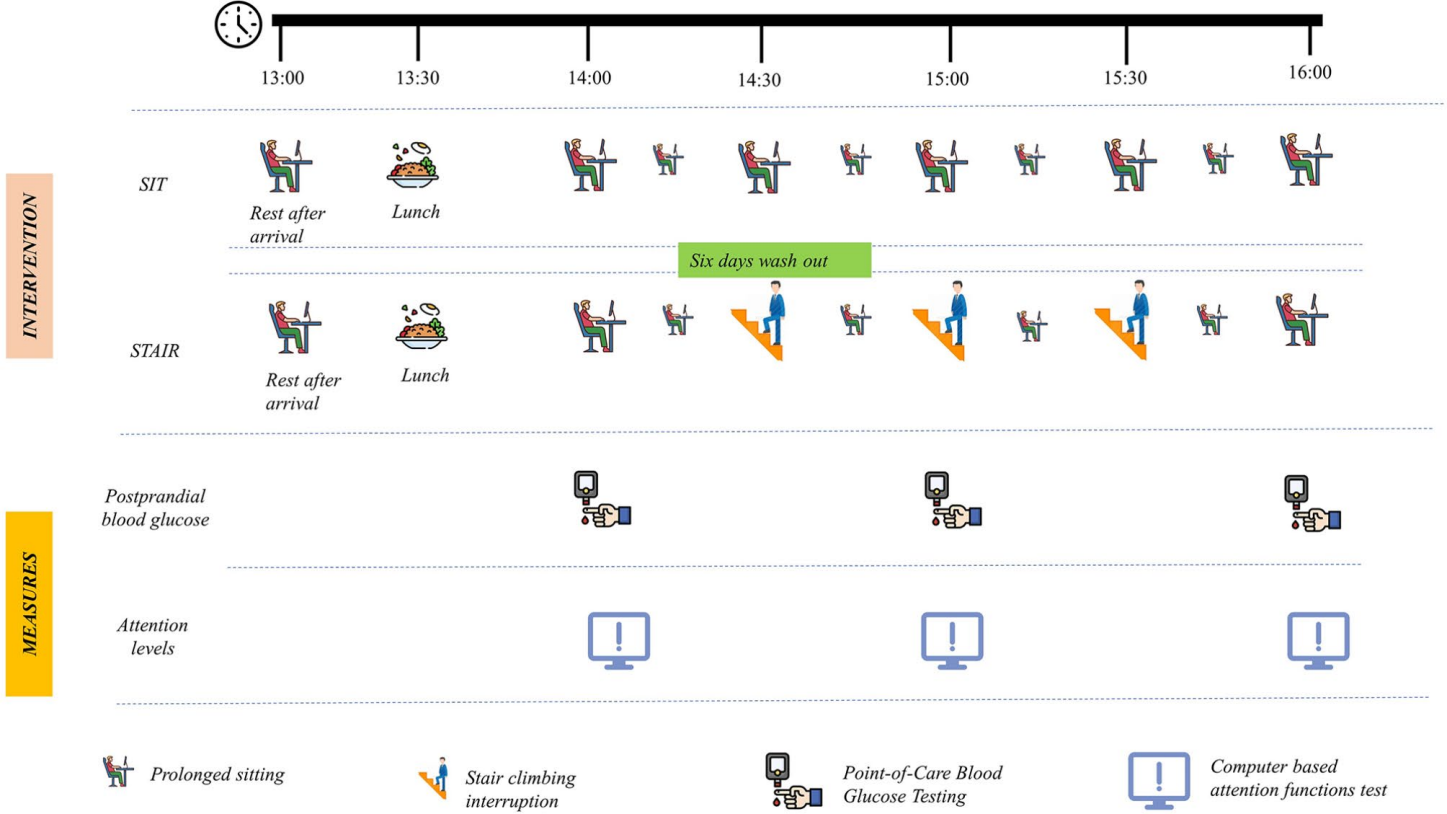
Procedure of the SIGMA trial. The participants underwent two visits: (1) SIT, uninterrupted sitting for two hours simulated work task; (2) STAIR, two minutes of stair climbing bouts every one 30 min of uninterrupted sitting for next two hours after a standardised lunch. The capillary blood glucose and attention levels were measured thrice during the visit: 0th minute, 60th minute and 120th minute of the intervention.

On the first visit, the participants were brought to the testing centre by the motorised vehicle and 50-meter walk from the parking zone. On arrival at 1:00 PM, the participants were seated in a standard workstation and were asked to watch videos for next 30 min until lunch. Then standardised meal constituting 33% total daily calories of the day - (carbohydrates – 51%, protein – 18%, and fat − 31%) were provided at 1:30 in the same workstation and the assigned intervention was started at 2:00 PM. The baseline capillary glucose and computer-based choice reaction times were assessed prior to the intervention. Based on the randomisation, the participants underwent either STAIR or SIT intervention during the first visit. If the participants have chosen STAIR intervention on the day first day, the participants interrupted their sitting time by two minutes of stair ascent and descent every 30 min from the time of post lunch with the timer overseen by a postgraduate in exercise science. The rate of stair ascent and descent were determined by the participants (self-paced) however ensured moderate intensity (rating of perceived exertion of 12–13). The outcome measures (capillary glucose and choice reaction time) were measured at the end of an hour of intervention just before the 2nd stair climb bout. At the end of two hours of the trial, the participants accumulated three bouts of two minutes moderate intensity stair climbing and the outcome measures were repeated at the end of last 30 min of sitting. During the SIT visit, the participants were administered similar work tasks and outcome measures in the sitting position without stair climbing interruptions. During both the visits, the participants were allowed only once to the restroom which was 30 feet away level surface from the testing area. The whole procedure is depicted in Fig. 5.

### Standardisation of work simulation tasks

To avoid the influence of the heterogenous tasks (seeing video, self-interested tasks) during the prolonged sitting on the cognitive functions, the tasks during the 2-hour trial were standardised. Participants were instructed to perform the following occupational simulating tasks consecutively in each position for two hours with 30 min each task: (1) copying text; (2) typing; (3) logical reasoning test; (4) drawing task. Each task is explained in the supplementary file S2. Each task was set to complete in 30 min. The four simulating tasks were randomised to avoid the cognitive influence across the intervention visits. Earlier experimental studies have administered tasks with low cognitive load such as reading, watching movies or non-stimulatory documentaries or using smartphones which lack ecological validity^40^.

### Outcome measures

#### Postprandial glycaemia

The primary outcome measure was postprandial glucose levels. Blood glucose levels were measured using the capillary method with a glucometer (Accu-Chek Active, Roche Diagnostics, USA). Tests were conducted immediately after the participant resumed simulated working conditions post-lunch, at the end of the 1st hour, and at the end of the 2nd hour when the trial concluded. The skin puncture site was cleaned with sterile cotton and disinfected with a 70% aqueous solution of isopropanol, following capillary blood sampling guidelines^41^. A lancing device was used to puncture the skin on the right index or middle fingertips, as per manufacturer standards. The first drop of blood was discarded using aseptic cotton, and the second drop was used for glucose analysis. If there was a measurement error (a difference of more than 1.0 mg/dL between the first two blood samples), an additional finger capillary sample was taken. The average of the two blood samples with the least variance was recorded as the glucose concentration (mg/dL) for that time point. The blood glucose was measured at 0th, 60th, and 120th minutes for both the STAIR and SIT trials.

#### Attention functions

Attention functions were measured using a choice reaction test based on the Deary-Liewald task^42^. This test assesses a participant’s reaction time to stimuli and typically takes 15–20 min per participant and measured at the last 15 min of each hour of the trial (Fig. 5). Participants sat in front of a computer screen and used their dominant hand to react to visual stimuli by pressing specific buttons on the keyboard corresponding to the stimuli displayed on the screen. Two tests were measured: (1) simple reaction and (2) choice reaction tests. For simple reaction tests, one square box with a cross mark within it was displayed and the participant must respond to stimuli at the earliest by pressing any key. The time taken to respond to the stimuli was accounted for the reaction times to the simple reaction test stimuli^42^. For the four-choice reaction time test, four horizontal stimuli (X marks within four small boxes) appear on the screen, and participants were instructed to press the button corresponding to the stimulus’s position^42^. They were instructed to press the z-key for the far-left box, the x-key for the second box from the left, the comma key for the second box from the right, and the period key for the far-right box. Reaction times are recorded in milliseconds. The relative reliability of this choice reaction test was rated as good to excellent (ICC = 0.89–0.93) for elderly populations with and without cognitive impairment^43^.

### Data analysis

All the statistical analysis was analysed using Jeffreys Amazing Statistical Package (version 0.18.1, University of Amsterdam, Netherlands). Normality was confirmed using Shapiro Wilk test. Last observation carried forward approach was followed for the data of two participants who missed the end follow up visit. The continuous measures (post prandial glucose levels, reaction times, accuracy) were described as mean ± standard deviation (SD) if variables are normalised and median (interquartile range) if not normalised. Linear mixed model analyses were used to gauge the effect of the time (0th, 1st and 2nd hr) and the condition (STAIR versus SIT) and their interaction effects on post prandial glucose levels, glucose AUC and attention functions (reaction times and accuracy to simple and choice reaction stimuli) as dependent variables while time (baseline, 1st hour and 2nd hour), condition (SIT and STAIR) as fixed variables and participant as a random factor with adjustment for baseline variability and imbalances in randomisation order. Models included fixed factors of condition, time and interaction (condition x time), controlled for baseline values and confounders (randomisation, age and gender). Estimation plots were also drawn to confirm the interaction and main effects of the outcomes. Further the association of change in glucose levels with the change in attention scores was explored using Pearson correlation tests. An alpha level of α ≤ 0.05 was set a priori for statistical significance for all the analyses. All data are presented as mean (95% CI) unless stated otherwise.

## Electronic Supplementary Material

Below is the link to the electronic supplementary material.

Supplementary Material 1

## Data Availability

The data used in the study is available with the corresponding author and will be made available based on the reasonable request to the corresponding author.
