# Supplementary material for: Short-term effects of brief stair climbing interruptions on postprandial hyperglycemia during prolonged sitting: a randomized cross-over trial
Source: Sci Rep. 2025 Jan 17;15:2329. doi: 10.1038/s41598-024-77827-3 (PMC11742412; doi:10.1038/s41598-024-77827-3)
Supplement: Supplementary file 1 — Supplementary Material 1 [file 41598_2024_77827_MOESM1_ESM.docx]

**Short-Term Effects of Brief Stair Climbing Interruptions on Postprandial Hyperglycemia During Prolonged Sitting: A Randomized Cross-Over Trial**

**Supplementary files**

Supplementary file S1: CONSORT 2010: extension to randomised cross-over trials.

Supplementary file S2. Standardisation of the computer-based tasks during the two hours of visit

Supplementary file **S1**: CONSORT 2010: extension to randomised cross-over trials.

| **Section and Topic** | **Item #** | **Checklist item** | **Location where item is reported** |
| --- | --- | --- | --- |
| **TITLE** | | |  |
| Title | 1a | Identification as a randomised crossover trial in the title | 1 |
| **ABSTRACT** | | |  |
| Abstract | 1b | Specify a crossover design and report all information outlined in CONSORT 2010 extension | 2 |
| **INTRODUCTION** | | |  |
| Rationale | 2a | Scientific background and explanation of rationale | 3 |
| Objectives | 2b | Specific objectives or hypotheses | 3-4 |
| **METHODS** | | |  |
| Trial design | 3a | Rationale for a crossover design. Description of the design features including allocation ratio, especially the number and duration of periods, duration of washout period, and consideration of carry over effect. | 7 |
| Change from protocol | 3b | Important changes to methods after trial commencement (such as eligibility criteria), with reasons | NA |
| Participants | 4a | Eligibility criteria for participants | 7 |
| Settings and location | 4b | Settings and locations where the data were collected | 5 |
| Interventions | 5 | The interventions with sufficient details to allow replication, including how and when they were actually administered | 9 |
| Outcomes | 6a | Completely defined prespecified primary and secondary outcome measures, including how and when they were assessed | 8-9 |
|  | 6b | Any changes to trial outcomes after the trial commenced, with reasons | No |
| Sample size | 7a | How sample size was determined, accounting for within participant variability | 8 |
| Interim analyses and stopping guidelines | 7b | When applicable, explanation of any interim analyses and stopping guidelines | NA |
| **Randomisation** | | | |
| Sequence generation‡  methods | 8a | Method used to generate the random allocation sequence | 10 |
|  | 8b | Type of randomisation; details of any restriction (such as blocking and block size) | 10 |
| Allocation concealment mechanism‡ | 9 | Mechanism used to implement the random allocation sequence§ (such as sequentially numbered containers), describing any steps taken to conceal the sequence until interventions were assigned | 10 |
| Implementation | 10 | Who generated the random allocation sequence,§ who enrolled participants, and who assigned participants to the sequence of interventions | NA |
| Blinding | 11a | If done, who was blinded after assignment to interventions (for example, participants, care providers, those assessing outcomes) and how | NA |
| Similarity of interventions | 11b | If relevant, description of the similarity of interventions | NA |
| Statistical methods | 12a | Statistical methods used to compare groups for primary and secondary outcomes which are appropriate for crossover design (that is, based on within participant comparison) | 11 |
| Additional analyses | 12b | Methods for additional analyses, such as subgroup analyses and adjusted analyses | 11 |
| **RESULTS** | | |  |
| Participant flow (a diagram is strongly recommended | 13a | The numbers of participants who were randomly assigned, received intended treatment, and were analysed for the primary outcome, separately for each sequence and period | Figure 1 |
| Losses and exclusions† | 13b | No of participants excluded at each stage, with reasons, separately for each sequence and period | Figure 1 |
| Recruitment‡ | 14a | Dates defining the periods of recruitment and follow-up | NA |
| Trial end | 14b | Why the trial ended or was stopped | Figure 1 |
| Baseline data† | 15 | A table showing baseline demographic and clinical characteristics by sequence and period | Table 1 |
| Numbers analysed | 16 | Number of participants (denominator) included in each analysis and whether the analysis was by original assigned groups | Figure 1 |
| Outcomes and estimation | 17a | For each primary and secondary outcome, results including estimated effect size and its precision (such as 95% confidence interval) should be based on within participant comparisons.¶ In addition, results for each intervention in each period are recommended | 4-5 |
| Binary outcomes | 17b | For binary outcomes, presentation of both absolute and relative effect sizes is recommended | NA |
| Ancillary analyseS | 18 | Results of any other analyses performed, including subgroup analyses and adjusted analyses, distinguishing prespecified from exploratory | 5 |
| Harms | 19 | Describe all important harms or untended effects in a way that accounts for the design (for specific guidance, see CONSORT for harms32) | NA |
| **DISCUSSION** | | |  |
| Limitations | 20 | Trial limitations, addressing sources of potential bias, imprecision, and if relevant, multiplicity of analyses. Consider potential carry over effects | 6 |
| Generalisability | 21 | Generalisability (external validity, applicability) of the trial findings | 6 |
| Interpretations | 22 | Interpretation consistent with results, balancing benefits and harms, and considering other relevant evidence | 6 |
| **OTHER INFORMATION** | | |  |
| Registration | 23 | Registration number and name of trial registry | Clinical trial registry of india (CTRI/2024/02/062247) |
| Protocol | 24 | Where the full trial protocol can be accessed, if available | NA |
| Funding | 25 | Sources of funding and other support (such as supply of drugs), role of funders | Manipal Academy of Higher Education Student seed grant - Title page |

Supplementary file **S2.** Standardisation of the computer-based tasks during the two hours of visit


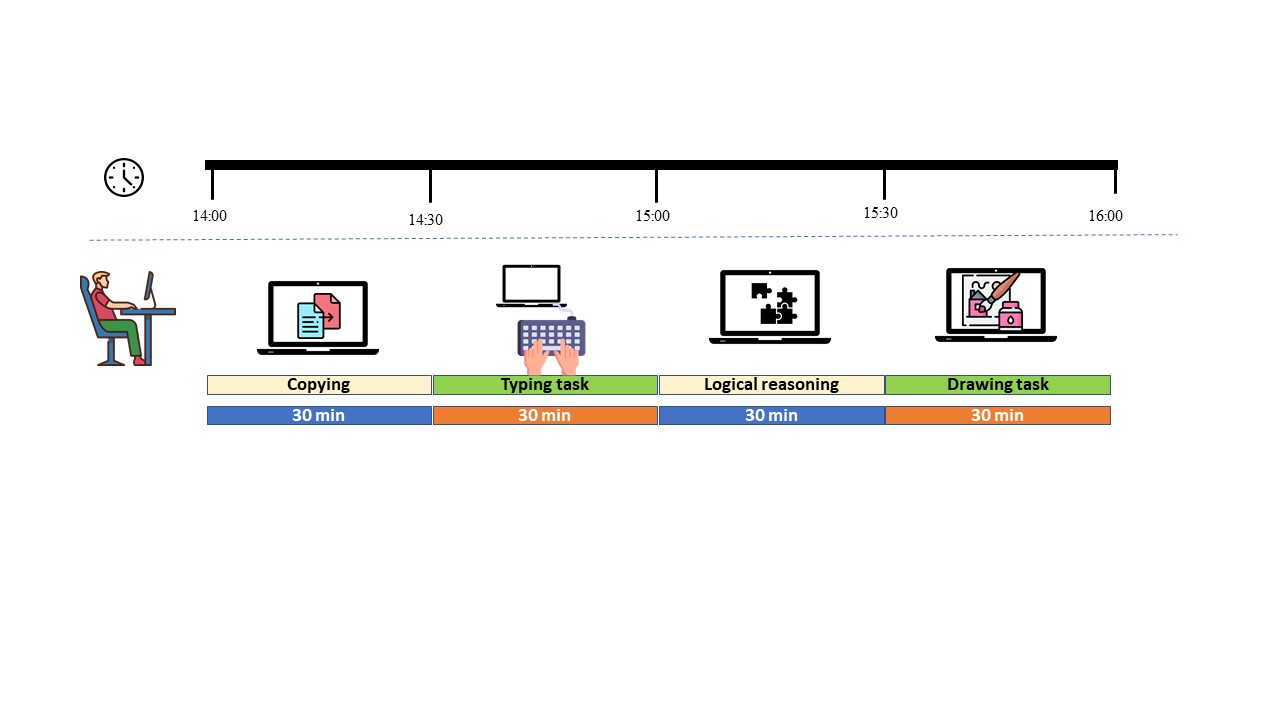


*30 minutes copying task:* For this task, the participants were instructed to type two-page report on two different regarding physical health (covered in sports science curriculum) on the two visits (1) physical fitness (<https://ncert.nic.in/textbook/pdf/iehp104.pdf>) on first visit; (2) IPC guide on Paralympic athletes (<https://www.paralympic.org/sites/default/files/2021-01/IPC%20Guide%20to%20Reporting%20on%20Para%20Athletes.pdf>) on second visit. The copying task lasted for 30 minutes.

*30 minutes typing speed test:* The participants were administered with an online typing task for 30 minutes in English (English typing speed test - <https://www.fast-typing.com/typing-speed-test.php?lan=english&time=1800>). The accuracy and word per minute were retrieved from the display at the end of the 30 minutes of the typing task.

*30 minutes logical reasoning:* During this non-verbal logical reasoning task, the participants were asked to choose a task for 30 minutes from the number of non-verbal reasoning tasks available from website such as mirror image, water images, paper folding (<https://www.indiabix.com/non-verbal-reasoning/questions-and-answers/>). The logical reasoning test comprised of 13-25 questions, each containing a grid of symbols or images. Each question had four possible answers, out of which one was correct. The participants were asked to choose the best options that appropriately fitted the symbols or question. The maximum time limit was set for 30 minutes.

*30 minutes of Drawing game:* This task was to simulate similar work condition of web designers. During this task, the participants were given ten blocks of drawing tasks where each block had six drawing tasks (Quick, draw! <https://quickdraw.withgoogle.com/>). The participants were asked to draw the object as directed within 30 seconds and the neural network, Google recognised the object drawn and match with the known objects. The number of correctly recognised objects were shown at the end of each block of six sets of drawings.
